# Impact of gynecologic cancer on pelvic floor disorder symptoms and quality of life: an observational study

**DOI:** 10.1038/s41598-019-38759-5

**Published:** 2019-02-19

**Authors:** Mathias Neron, Sophie Bastide, Renaud de Tayrac, Florent Masia, Catherine Ferrer, Majd Labaki, Laurent Boileau, Vincent Letouzey, Stephanie Huberlant

**Affiliations:** 1 Department of Obstetrics and Gynecology, Nîmes University Hospital, Univ., Montpellier, Nîmes France; 2 Department of BESPIM (Biostatistics, Epidemiology, Public Health and Innovation in Methodology), Nîmes University Hospital, Univ., Montpellier, Nîmes France

## Abstract

The objective of our observational prospective study was to investigate the severity and prevalence of urinary and pelvic floor disorders in gynecologic cancer survivors. All patients surviving gynecological cancer in the region as well as women receiving invitations to attend breast-screening checkups as the control population were asked to fill-in questionnaires assessing pelvic prolapse symptoms (PFDI-20, Wexner) and associated quality of life (PFIQ-7). Eighty-nine women were included in the cancer survivor group and 1088 in the control group. Pelvic floor symptoms (PFDI-20 questionnaire) were significantly worse in cancer survivors than in control women (score: 33.3 [14.6–74.1] vs. 20 [4.2–50.0], *p* = 0.0003). Urge incontinence was significantly worse in cancer survivors in both univariable (ORb = 2.061 [95% CI = 1.284–3.309], *p* = 0.0027) and multivariable analyses (ORa = 1.672 [95% CI = 1.014–2.758], *p* = 0.0442), as was fecal incontinence in univariable (ORb = 3.836 [95% CI = 1.710–8.602], *p* = 0.0011) and in multivariable (ORa = 3.862 [95% CI = 1.657–9.001], *p* = 0.0018) analyses. Women with benign hysterectomies had poorer quality of life and increased pelvic floor disorders compared to women with no history of surgery. Survivors of gynecological cancer experience significantly more pelvic floor symptoms and an associated reduction in quality of life.

## Introduction

Cancer incidence increases continuously in developed countries as the mean age of the overall population rises. The number of patients living with cancer and surviving treatment is also rising, tripling over the last three decades to reach more than 10 million survivors in the United States alone^1–4^. Constantly improving cancer treatments have increased survival rates in almost all types of cancer^5^. However, important differences are observed in cancer care according to the cancer type and location, such as screening and treatments, and in follow-up and survival^6^.

With an annual gynecologic malignancies incidence of 15,000 in France in 2012^7^ and more than 96,000 in the United States in 2017^8^, ovarian, endometrial, and cervical cancers are a major cancer group in women. Treatment options for these malignancies combine radiation therapy, chemotherapy and varying degrees of radical surgical procedures, resulting in an overall 5-year survival rate of 70% in the United States^8^. In view of the advances in these gynecologic cancer treatments, it is thus essential to assess treatment side-effects in these patients. Indeed, the common treatment strategies often leads to long-term side-effects, including those related to urinary and pelvic floor disorders (PFD), and impact the quality of life of women surviving gynecologic cancers. Pelvic and urinary disorders include pelvic organ prolapse, urinary incontinence (stress and urge) and fecal incontinence. They have become a major public health issue in developed countries, and quality of life associated with PFD is now considered of a major importance for these patients^9–11^.

When evaluating PFD, the different treatment approaches (sole or combined treatments) may be confounding factors of pelvic disorder sequelae^12,13^. Also, cancer itself may be an independent confounding factor in evaluating PFD in survivors, because these symptoms are often more tolerated, if not totally neglected, by many cancer survivors^14,15^. Previous studies have assessed or reviewed issues related to specific treatment types or specific cancer types^16–21^, although few have assessed these aspects in cohort studies^22,23^. The impact of hysterectomy on PFD is still debated, studies showing conflicting results^9,10^. Data on symptoms and quality of life of gynecologic cancer survivors are thus still insufficient.

The main objective of the present study was to assess the prevalence of pelvic floor, urinary and fecal disorders in gynecologic cancer surviving patients compared to the general population through a self-questionnaire (PFDI-20). Secondary objectives were (i) to evaluate the effects of these symptoms on quality of life through another self-questionnaire (PFIQ-7) and (ii) to compare, in a subgroup analysis, the prevalence of these symptoms and their effect on cancer survivors quality of life in women who underwent benign hysterectomy and women without history of cancer or hysterectomy.

## Methods

### Study design and setting

This observational prospective cross-sectional study was divided into four stages: (i) information campaign via local radio and newspapers, (ii) mailing of the questionnaires, (iii) data collection and (iv) data analysis. The study was conducted over a 12-month period. It was approved by the Institutional Review Board (IRB 13/02-02). No signed informed consent was required as all questionnaires were anonymous; however, patients had one month after returning the questionnaire to withdraw their participation before data were used.

This study was conducted in the Obstetrics and Gynecology department of a tertiary University Hospital in France, for cancer survivor enrolment, and in the local region for control population enrolment.

### Participants

The cancer survivors (CS) group gathered gynecologic (ovarian, endometrial, cervical) cancer patients treated at the gynecologic cancer Department of our University Hospital. Patients were considered survivors and included in the survivors group if they were in remission and treatment-free for at least one year before enrolment. Control women were representative of the regional general population and were enrolled through an anonymous questionnaire sent along with the systematic biannual invitation for breast cancer screening by the Gard-Lozere Cancer Screening Program. This screening program invites all French women between 50 and 74 years old to perform a mammography, therefore its participants are well representative of this population. Women recruited in both groups were aged between 50 and 75 years old at the time the questionnaires were sent. CS and control women received the same questionnaires. CS were recruited between October 2013 and June 2014 and control women between October 2013 to April 2014.

For secondary analysis, the control group was divided into two subgroups: (i) women with history of simple hysterectomy for benign disease (CGH^+^) and (ii) women with neither history of pelvic surgery nor cancer (CGH^−^). We chose to perform this subgroup analysis due to the lack of data on the role of hysterectomy on PFD incidence in the literature^9,10^.

### Variables

The survey included general demographic and medical information, as well as three international self-questionnaires both validated in French, the PFDI-20 questionnaire for assessment of PFD and urinary symptoms as well as pelvic pain, the PFIQ-7 questionnaire for assessment of PFD effects on quality of life and the Wexner questionnaire for assessment of fecal incontinence. The PFDI-20 consists in 20 questions on urinary (six questions), digestive (eight questions) and pelvic prolapse (six questions) symptoms. Each specific question evaluates the presence of the symptom (0- absence of the symptom) and the functional discomfort caused (from 1- “not at all” to 4- “quite a bit”). Some questions are used as stand-alone responses to assess specific symptoms (question 3 for prolapse, question 16 for urge incontinence, and question 17 for stress incontinence). The PFIQ-7 consists in seven questions on the effects on daily activities of three groups of symptoms (Bladder or urine, Bowel or rectum, Vagina or pelvis), using the same 4-point scale of frequency than the PFDI-20 questionnaire. They assess the social impact of urinary and digestive symptoms, and prolapse. The Wexner questionnaire was used preferably to the fecal incontinence questions from the PFDI-20 questionnaire, as we considered it was more extensive and better targeted. It assessed frequency and disruptiveness of fecal incontinence using five questions with a 5-point scale (0- “never” to 5- “always”).

### Statistical methods

#### Data sources

A database was created manually and validated with datascan by the biostatistics department of our university hospital. It included all completed questionnaires returned. Telephone calls were made to complete missing data for CS patients.

#### Bias

To reduce the geographic bias, women from a unique region were recruited and sampling bias was reduced by sampling the population.

#### Sample size

For the CS group, the expected response rate was of 50%; three phonecalls were made to achieve this rate. For the control group, as most questionnaire surveys have response rates between 15% and 20%^24,25^, our expected response rate was set-up at 10%. Thus, to obtain 1000 responses, 10000 questionnaires were sent, which required 2.5 months of screening program mailing, sent over a3-month period to ensure the feasibility of the study.

As there were no previous studies on the subject, we have considered prevalence data to calculate our sample size, considering that the intensity of the symptoms is correlated with the prevalence reported. Prevalence of urinary incontinence is about 20% in women above 50 years old in the general population^26^, around 40% in gynecologic cancer survivors^23^, and unknown in patients with hysterectomy for benign disease. To detect a difference of 20%, with an alpha error of 5% and a beta error of 90% in case control comparison, and an odds ratio of 2 with a beta error of 80% in comparison with hysterectomy for benign disease group, with a 1:3 ratio for case control, and a 1:1 ratio for the subgroup analysis, 100 cancer survivors and 400 control patients with completed returned questionnaires were required.

#### Analysis

The population was described with continuous quantitative data expressed as means and standard deviations (SD). The two groups were compared using the t-test. Discrete quantitative data are presented as medians with their 25^th^ and 75^th^ percentiles, and compared using the Mann Whitney-U test. Qualitative variables are expressed as frequencies with percentages and compared using the chi-square test (or Fisher exact test if necessary). The global PFDI-20 score (primary outcome) and the global PFIQ-7 score (secondary outcome) were analyzed using the same methods for both two-groups and three-groups. The association between the global score and the group was first explored using the Mann Whitney-U test for two groups and the Kruskal–Wallis test for three groups, and were presented graphically (boxplot).

A regression analysis was performed to assess a possible association; potential cofactors, especially variables associated with PFD incidence (age, menopause treatment) and variables unbalanced between groups, were taken into account. Because of the non-Gaussian distribution with overdispersion and the high proportion of “0” in the questionnaires’ responses, the Zero-inflated Negative Binomial (ZINB) model was used.

The ZINB model is divided into two stages: (i) a logistic step to model the excess zeros binary process and (ii) a negative binomial stage to model the count process with overdispersion. Univariable and multivariable analyses were performed. The results are presented as crude (cOR) and adjusted (aOR) odds ratios for the logistic stage and as crude (cIRR) and adjusted (aIRR) Incidence Rate Ratios for the negative binomial stage of the univariable and multivariable analyses, respectively. All estimations are presented with their 95% Confidence Intervals (95% CI). For model interpretation, a risk factor (more symptoms with higher PFDI-20 score or more discomfort with higher PFIQ-7 score) was associated with an OR >1 (increased risk of having a score >0) and/or an IRR > 1 (increase value of the score even non-equal to 0). Although originally planned in the study protocol, individual age matching (±five years) between the groups was not performed to use the entire population as the age difference between the two groups was of only two years.

All analyses were performed using SAS version 9.4 (SAS Institute Inc., Cary, NC), with a 2-sided type 1 error rate of 5% as threshold for statistical significance.

### Details of Ethics Approval

This study was approved by Institutional Review Board of Nimes University. Date of approval: 2012, February (IRB 13/02-02).

## Results

### Participants’ characteristics

**Figure 1:**
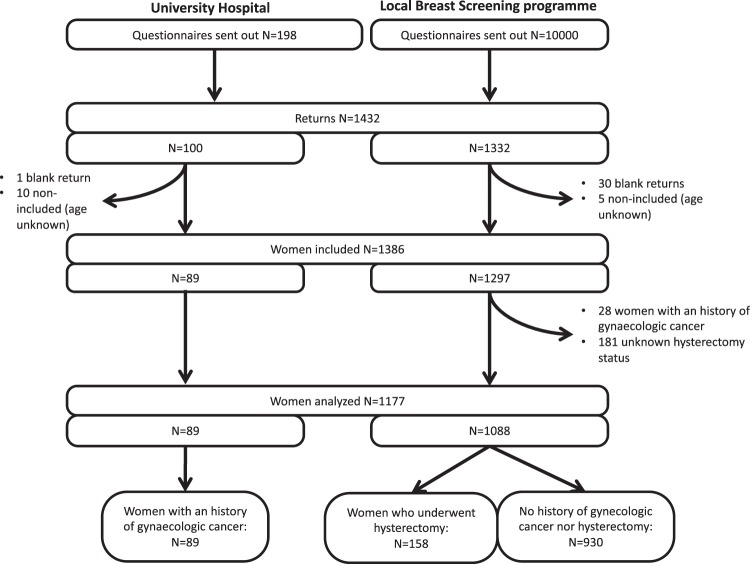
Flow chart.

Questionnaires from 1,386 patients were included, of which 1,177 were analyzed (correctly completed): 89 from the CS group and 1,088 from the control group (Fig. 1, study flowchart). Matching was not deemed useful because of the similarity between the groups, therefore, to maximally exploit all the data available, all patients from the control group with correctly completed questionnaires were included in the analysis.

Baseline participant characteristics are shown in Table 1. Briefly, CS women were older (63.73 ± 6.46 *vs*. 61.69 ± 6.84, *p* .80.0073) and had a higher BMI (27.38 ± 7.4 *vs*. 25.07 ± 4.89, *p* .80.0056) than women in the control group. All CS underwent a hysterectomy as part of the treatment of their gynecological cancer. Baseline data of analyzed and non-analyzed women of both cancer survivors and control groups were compared. Overall, non-analyzed women of the control group were younger, with lower BMI, and under hormonal menopause treatment (data not shown).

**Table 1:** Participant characteristics.

	CS Group		Control group		P-values
	*MD*	N = 89	*MD*	N = 1269	
Age	*2*	63.72 ± 6.46	*13*	61.69 ± 6.84	0.0073^†^
Weight (kg)	*2*	70.89 ± 18.27	*11*	65.46 ± 13.00	0.0078^†^
Height (cm)	*0*	161.26 ± 5.73	*9*	161.60 ± 5.87	0.5946^†^
BMI (kg/m²)	*2*	27.36 ± 7.40	*18*	25.07 ± 4.89	0.0056^†^
Parity	*2*	2 [1;3]	*12*	2 [1;3]	0.4916^‡^
Birth weight of the biggest child (g)	*8*	3458 ± 729	*42*	3447 ± 541	0.9020^†^
Professional status:	*2*		*23*		0.1853*
Active		20 (22.99%)		404 (32.43%)	
Retired		55 (63.22%)		684 (54.90%)	
Other		12 (13.79%)		158 (12.68%)	
Menopausal hormone replacement therapy:					
Vaginal Gel or ovules	*18*	8 (11.27%)	*168*	146 (13.26%)	0.6299*
Percutaneous gel or daily pills	*7*	22 (26.83%)	*95*	283 (24.11%)	0.5781*
History of breast cancer	*13*	9 (11.84%)	*93*	49 (4.17%)	0.0065^#^
History of POP surgery	*25*	3 (4.69%)	*176*	53 (4.85%)	0.9533*

MD: missing data; POP: pelvic organ prolapse.

Results are expressed as: n (%); mean ± standard deviation; median [inter-quartile range].

*Khi² test; ^#^Exact Fisher test; ^†^Student t-test; ^‡^Mann-Whitney test.

### PFDI-20

**Figure 2:**
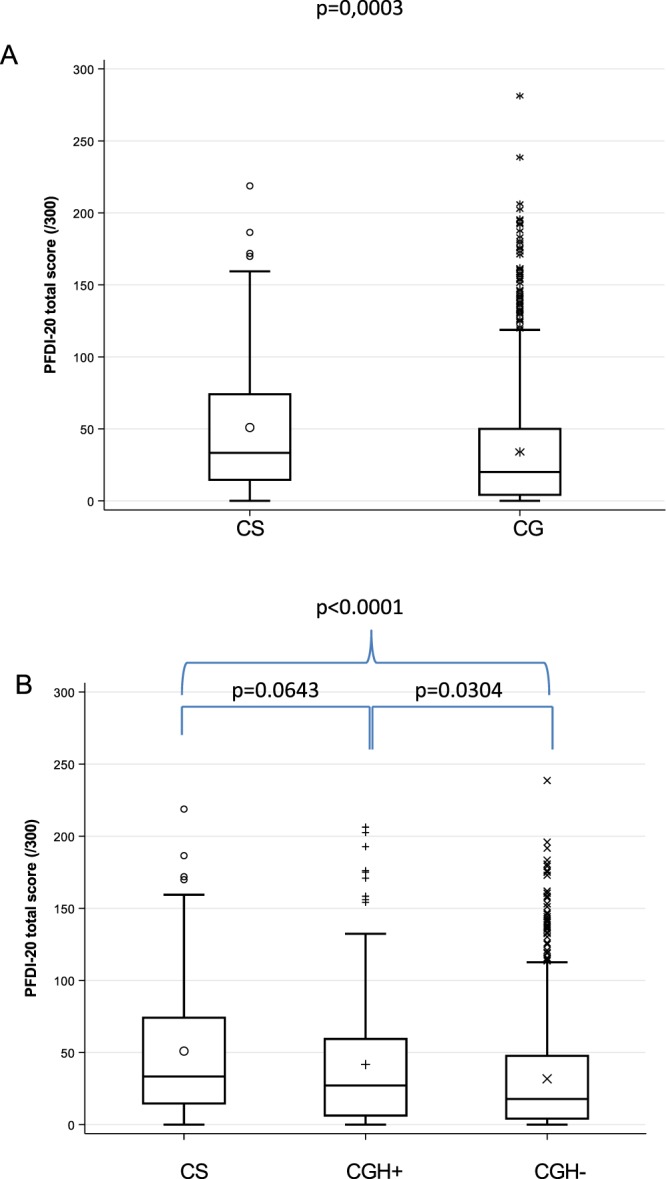
Boxplot comparing PFDI-20 global score of (**A**) cancer survivor (CS) to control group (CG) and (**B**) cancer survivors (CS) to women who underwent benign hysterectomy (CGH^+^) and women with no history of cancer or hysterectomy (CGH^−^). Global score ranges from 0 to 300, higher scores represent more symptoms and associated discomfort.

The nonparametric univariable analysis of the PFDI-20 global score showed a significant difference between CS and control women (score: 33.3 95% CI [14.6–74.1] *vs*. 20 95% CI [4.2; 50.0], *p* = 0.0001) (Fig. 2A). Adjustment on potential confounding factors was performed by univariable and multivariable analyses using the ZINB model (Table 2). CS showed a significantly worse score in both the logistic part and the negative binomial part of the model for univariable analysis, and in the negative binomial part of the model in multivariable analysis (aIRR: 1.459 [95% CI: 1.110–1.934], *p* = 0.0058). The logistic part of the model was not significant in multivariable analysis.

**Table 2:** Univariable and multivariable analyses comparing global PFDI-20 score of two groups: women with (CS) and without history of gynecological cancer (CG); and three groups: women with history of gynecological cancer (CS), women who underwent benign hysterectomy (CGH+) and women without history of gynecological cancer or hysterectomy (CGH−) with Zero-inflated Negative Binomial (ZINB) model.

Logistic part of the model	Univariable analysis			Multivariable analysis		
	cOR	CI 95%	p-values	aOR	CI 95%	p-values
**Two-groups analysis (N = 909 final multivariable model)**						
Group:						
Women w/o pelvic cancer	1			1		
Women with pelvic cancer	3.006	[1.099; 8.226]	0.0094	2.214	[0.789; 6.210]	0.0899
**Negative binomial part of the model**	**cIRR**	**CI 95%**	**p-values**	**aIRR**	**CI 95%**	**p-values**
Group:						
Women w/o pelvic cancer	1			1		
Women with pelvic cancer	1.323	[1.046; 1.673]	0.0153	1.459	[1.110; 1.934]	0.0058
**Three-groups analysis (N = 914 final multivariable model ¥)**						
**Logistic part of the model**	**cOR**	**CI 95%**	**p-values**	**aOR**	**CI 95%**	**p-values**
Group:			0.0222			0.1294
Women w/o pelvic cancer	1			1		
Women with hysterectomy	1.130	[0.687; 1.859]	0.6302	1.072	[0.619; 1.854]	0.8049
Women with pelvic cancer	3.174	[1.170; 8.606]	0.0233	2.388	[0.916; 6.224]	0.0749
*Women with pelvic cancer vs. women with hysterectomy*	*2.809*	*[0.950; 8.309]*	*0.0620*	*2.229*	*[0.766; 6.486]*	*0.1414*
**Negative binomial part of the model**	**cIRR**	**CI 95%**	**p-values**	**aIRR**	**CI 95%**	**p-values**
Group:			0.0011			0.0056
Women w/o pelvic cancer	1			1		
Women with hysterectomy	1.284	[1.062; 1.553]	0.0099	1.215	[0.989; 1.492]	0.0640
Women with pelvic cancer	1.402	[1.108; 1.773]	0.0049	1.436	[1.107; 1.862]	0.0064
*Women with pelvic cancer vs. women with hysterectomy*	*1.091*	*[0.821; 1.450]*	*0.5477*	*1.182*	*[0.866; 1.615]*	*0.2921*

COR: crude Odds Ratio; aOR: adjusted Odds Ratio; cIRR: crude Incidence Rate Ratio; aIRR: adjusted Incidence Rate Ratio; CI 95%: 95% confidence interval.

The multivariable models were adjusted for the 2-group and the 3-group analysis on the following cofactors respectively: BMI, treatment for vaginal dryness, and previous history of prolapse surgery (¤); BMI and treatment for vaginal dryness (¥). Variables tested but not retained in the final model: age, working status, social-professional category, treatment with hormonal replacement therapy and previous history of breast cancer.

For model interpretation, a risk factor (more symptoms with higher PFDI-20 score) is associated with an OR >1 (increased the risk of having a score > 0) and/or an IRR > 1 (increased the value of the score if >0).

### Stand-alone questions and Wexner questionnaire

Looking into the details of the key stand-alone questions of the PFDI-20 questionnaire showed that symptoms of stress incontinence were statistically worse in CS compared to controls in univariable analysis (ORb = 1.750 [95% CI = 1.111–2.755], *p* = 0.0158), but not in multivariable analysis (ORa = 1.510 [95% CI = 0.942–2.421], *p* = 0.0869). The only variable retained for multivariable analysis was BMI. Urge incontinence was significantly different between the two groups in both univariable and multivariable analyses, (ORb = 2.061 [95% CI = 1.284–3.309], *p* = 0.0027, and ORa = 1.672 [95% CI = 0.014–2.758], *p* = 0.0442, respectively). The only variables retained in the multivariable analysis were age and BMI. Prolapse was significantly more prevalent in the CS group in univariable analysis (5.88% *vs*. 0%, *p* = 0.0296). Multivariable analysis could not be performed on this dataset due to the data distribution. The Wexner score showed significantly more symptoms of fecal incontinence in CS than in control women in univariable analysis (ORb = 3.836 [95% CI = 1.710–8.602], *p* = 0.0011) and in multivariable analysis (ORa = 3.862 [95% CI = 1.657–9.001], *p* = 0.0018). The only variable retained for multivariable analysis was the number of children.

### PFIQ-7

**Figure 3:**
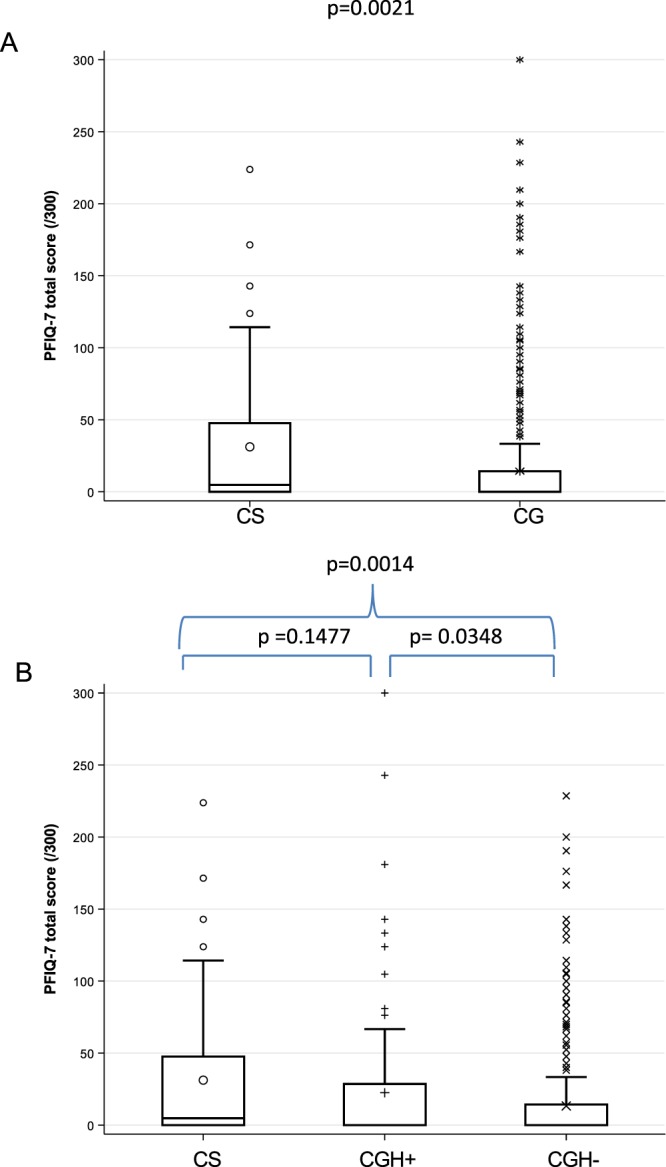
Boxplot comparing PFIQ-7 global score of (**A**) cancer survivor (CS) to control group (CG) and (**B**) cancer survivors (CS) to women who underwent benign hysterectomy (CGH^+^) and women with no history of cancer or hysterectomy (CGH^−^). Global score ranges from 0 to 300, higher scores represent a stronger impact of symptoms on quality of life.

The effect of the pelvic floor symptoms on quality of life as assessed by the global PFIQ-7 questionnaire was analyzed in nonparametric univariable analysis, showing a significant difference between the CS and the control groups (score: 4.8 95 CI% [0;47.6] *vs*. 0 95% CI [0;14.3], *p* = 0.0021) (Fig. 3A). Adjustment on potential confounding factors was performed in univariable and multivariable analyses using the ZINB model (Table 3). CS showed a significantly worse score in both the logistic part and the negative binomial part of the model for univariable analysis, but the score was not significantly different in either model in multivariable analysis.

**Table 3:** Univariable and multivariable analyses comparing global PFIQ-7 score of two groups: women with (CS) and without history of gynecological cancer (CG);; and three groups: women with history of gynecological cancer (CS), women who underwent benign hysterectomy (CGH+) and women without history of gynecological cancer or hysterectomy (CGH−) with Zero-inflated Negative Binomial (ZINB) model.

Logistic part of the model	Univariable analysis			Multivariable analysis		
	cOR	CI 95%	p-values	aOR	CI 95%	p-values
**Two-groups analysis (N = 880 final multivariable model)**						
Group:						
Women w/o pelvic cancer	1			1		
Women with pelvic cancer	1.928	[1.114; 3.338]	0.0164	1.788	[0.966; 3.310]	0.0594
**Negative binomial part of the model**	**cIRR**	**CI 95%**	**p-values**	**aIRR**	**CI 95%**	**p-values**
Group:						
Women w/o pelvic cancer	1			1		
Women with pelvic cancer	1.601	[1.109; 2.313]	0.0076	1.410	[0.959; 2.075]	0.0681
**Three-groups analysis (N = 822 final multivariable model ¥)**						
**Logistic part of the model**	**cOR**	**CI 95%**	**p-values**	**aOR**	**CI 95%**	**p-values**
Group:			0.0348			0.0740
Women w/o pelvic cancer	1			1		
Women with hysterectomy	1.250	[0.847; 1.845]	0.2601	1.201	[0.779; 1.851]	0.4065
Women with pelvic cancer	1.957	[1.128; 3.395]	0.0169	1.956	[1.061; 3.605]	0.0315
*Women with pelvic cancer vs. women with hysterectomy*	*1.565*	*[0.825; 2.970]*	*0.1704*	*1.629*	*[0.799; 3.322]*	*0.1798*
**Negative binomial part of the model**	**cIRR**	**CI 95%**	**p-values**	**aIRR**	**CI 95%**	**p-values**
Group:			0.0005			0.0231
Patients without medical history	1			1		
Patients with previous hysterectomy	1.506	[1.124; 2.016]	0.0060	1.313	[0.964; 1.788]	0.0844
Patients with history of gynecological cancer	1.716	[1.191; 2.418]	0.0038	1.546	[1.056; 2.262]	0.0250
Patients with history of gynecological cancer *vs*. Patients with previous hysterectomy	*1.140*	*[0.736; 1.764]*	*0.5583*	*1.178*	*[0.745; 1.861]*	*0.4838*

COR: crude Odds Ratio; aOR: adjusted Odds Ratio; cIRR: crude Incidence Rate Ratio; aIRR: adjusted Incidence Rate Ratio; CI 95%: 95% confidence interval.

The multivariable models were adjusted for the 2-group and the 3-group analysis on the following cofactors respectively: BMI, working status, and treatment for vaginal dryness (¤); BMI, working status and treatment for vaginal dryness (¥). Variables tested but not retained in the final model: age, social-professional category, treatment with hormonal replacement therapy, previous history of breast cancer, and previous prolapse surgery.

For model interpretation, a risk factor (more discomfort with higher PFIQ-7 score) is associated with an OR >1 (increased the risk of having a score > 0) and/or an IRR > 1 (increased the value of the score if >0).

### Three-group comparison

To better understand the impact of cancer and cancer-independent hysterectomy on prolapse symptoms and on quality of life, we undertook a complementary analysis of both questionnaires aimed to compare CS to two subgroups of the control group: women without history of cancer but with history of benign hysterectomy (CGH+) and women without history of cancer or hysterectomy (CGH−).

For the PFDI-20 questionnaire, the nonparametric univariable analysis showed a significant difference between the three groups (*p* = 0.0001). CS women presented more symptoms (score: 33.3 [95% CI = 14.7–74.1]) as compared to CGH+ women (27.1 [95% CI = 6.3–59.4], *p* = 0.0643), who in turn had significantly more symptoms compared to CGH− women (17.7 [95% CI = 4.2–47.6], *p* = 0.0304) (Fig. 2B). Regarding the PFIQ-7 questionnaire, the nonparametric univariable analysis also showed a significant difference among the three groups (global p-value = 0.0013). PFD had a stronger effect on the quality of life of CS (score: 4.8 [95% CI = 0–47.6]) than of CGH− women (0 [95% CI = 0–14.3], *p* = 0.0014), and a trend was reported for compared to CGH+ women (0 [95% CI = 0–28.6], *p* = 0.1477) (Fig. 3B). The PFD effect on quality of life was significantly worse for CGH+ women as compared to CGH− women (*p* = 0.0348). The ZIMB model was used to analyze PFDI-20 and PFIQ-7 three-group comparisons. PFDI-20 and PFIQ-7 scores were significantly worse for CS women compared to CGH− women in the two parts of the model, but not compared to CGH+ women. Detailed results of the univariable and multivariable analyses are shown in Table 2 (PFDI-20) and Table 3 (PFIQ-7).

## Discussion

### Main Findings

Our study shows, for the first time, a correlation between occurrence of a gynecologic cancer and PFD symptoms. Women surviving of a gynecologic cancer had significantly more pelvic floor symptoms compared with women of the general population, including women having undergone benign hysterectomy. Hysterectomy did have an impact on pelvic floor symptoms and quality of life, but this impact was not as significant as cancer. It is important to keep in mind that all patients included in our study were in remission and treatment-free for more than one year. Therefore, symptoms described are not linked to an active disease but to the past disease and its treatments. Symptoms of fecal and urge incontinence and prolapse were also significantly worse in cancer survivors than the general population.

The interpretation of studies using questionnaires often comprises the description of the minimum clinically-important difference (MCID). However, this parameter does not seem adapted for epidemiological studies in almost only non-complaining women. The MCIDs for the PFDI-20 and PFIQ-7 questionnaires were set in 2005 at 46 (15%) and 36 (12%) points, in surgical cases of pelvic organ prolapse. Later, the MCID for PFDI-20 was set at 13.5 points in a population affected by PFD but treated conservatively. This threshold is very close to the difference of 13.3 points reported here, that could be considered significant and clinically relevant. No recent publications on MCID and PFIQ-7 were found. Due to the low level of symptoms burden, expected in this observational study in non-complaining women, the impact on quality of life and PFIQ-7 is very low (4.8 points). Also, we should consider that CS have undergone heavy treatments; their symptoms are probably reported in the perspective of their cancer history, and moderate their impact on their quality of life. However, although significant, the difference reported for the PFIQ-7 responses is difficult to interpret.

To date, few studies have investigated the impact of a history of gynecologic cancer on PFD symptoms and quality of life^22^. Indeed, previous studies have mainly focused on urinary symptoms and sexual dysfunction. Rutledge *et al*. found that urinary incontinence and pelvic organ prolapse did not differ between cancer survivors and controls, however, their study control group included patients under active gynecologic consultation. Nevertheless, they found a negative effect on sexual functions among cancer survivors as also reported in other studies^27^. A recent systematic review concluded that PFD, including urinary, fecal and sexual disorders, affected women three times more after than before their gynecologic cancer^28^. Our results suggest that, in addition to an increased prevalence, the severity of the PFD symptoms is higher in CS than in controls.

### Strengths and Limitations

The strengths of our study include the use of validated questionnaires and a large cohort of over 1000 cancer-free women as a control group. These women were recruited through a breast cancer screening program and not in gynecological consultations as in the majority of studies, and are more representative of the general population. To account for any differences between CS and controls, multivariable analyses were adjusted on potential confounding factors including BMI, working status, menopause treatment and history of breast cancer. A limitation of our study was that the CS treatments were not collected; therefore sound conclusions on the impact of radiotherapy on urogynecological symptoms cannot be made. We were not able to report initial characteristics on the neoplasia, including the primary site. This lack of data enables us to detect confounding factors associated with urogynecological symptoms development.

### Interpretation

From these results we can infer that the effect of hysterectomy is minimal on quality of life and on gynecological symptoms as compared to the cancer itself. It is also clear that CS have urogynecological symptoms that require active investigation during follow-up. We also establish a valuable role for the use of questionnaires in eliciting such information, which could easily be incorporated into the routine follow-up of these patients.

Further investigations on the effect of radiation or other therapies on pelvic organ prolapse symptoms and their consequences on the survivors’ quality of life still has to be assessed. Indeed, it is not possible at this point to determine whether the symptoms might be due to the therapy itself. Also, importantly, long-term adverse events of gynecologic cancers and treatments are often urogynecological events. These last years, the awareness of fertility in young women has improved and has conducted to the setting–up, in France, of specialized networks^29^. Likewise, our results should encourage oncologists to initiate discussions about pelvic floor disorders with gynecological cancer patients.

Based on these results, implementing a better follow-up of gynecologic cancer survivors affected by wide-ranging symptoms, yet who seemingly do not seek help, should be considered^15^. Future work should focus on determining the specific effects of the individual types of cancer and treatment of these symptoms in prospective studies. A comprehensive analysis of the sub-scores of the PFDI-20 questionnaire (Urinary Distress Inventory, Pelvic Organ Prolapse Distress Inventory and Colorectal-Anal Distress Inventory) will be the subject of a future analysis by our group.

## Conclusion

Our study highlights the effects of gynecological cancer on PFD, with worse scores of urge incontinence, fecal incontinence and quality of life in CS than in controls. The likely role of hysterectomy on PFD is again raised in this large cohort and should be considered in future research on this topic.
